# The emerging role of oral microbiota in oral cancer initiation, progression and stemness

**DOI:** 10.3389/fimmu.2023.1198269

**Published:** 2023-10-26

**Authors:** Partha Jyoti Saikia, Lekhika Pathak, Shirsajit Mitra, Bikul Das

**Affiliations:** ^1^ Department of Cancer and Stem Cell Biology, KaviKrishna Laboratory, Research Park, Indian Institute of Technology, Guwahati, India; ^2^ Department of Stem Cell and Infectious Diseases, KaviKrishna Laboratory, Research Park, Indian Institute of Technology, Guwahati, India; ^3^ Department of Experimental Therapeutics, Thoreau Laboratory for Global Health, M2D2, University of Massachusetts, Lowell, MA, United States

**Keywords:** oral squamous cell carcinoma (OSCC), stemness, oral leukoplakia (OLK), epithelial mesenchymal transition (EMT), altruistic stem cells (ASCs), cancer stem cells (CSCs)

## Abstract

Oral squamous cell carcinoma (OSCC) is the most prevalent malignancy among the Head and Neck cancer. OSCCs are highly inflammatory, immune-suppressive, and aggressive tumors. Recent sequencing based studies demonstrated the involvement of different oral microbiota in oral cavity diseases leading OSCC carcinogenesis, initiation and progression. Researches showed that oral microbiota can activate different inflammatory pathways and cancer stem cells (CSCs) associated stemness pathways for tumor progression. We speculate that CSCs and their niche cells may interact with the microbiotas to promote tumor progression and stemness. Certain oral microbiotas are reported to be involved in dysbiosis, pre-cancerous lesions, and OSCC development. Identification of these specific microbiota including *Human papillomavirus* (HPV), *Porphyromonas gingivalis* (PG), and *Fusobacterium nucleatum* (FN) provides us with a new opportunity to study the bacteria/stem cell, as well as bacteria/OSCC cells interaction that promote OSCC initiation, progression and stemness. Importantly, these evidences enabled us to develop *in-vitro* and *in-vivo* models to study microbiota interaction with stem cell niche defense as well as CSC niche defense. Thus in this review, the role of oral microbiota in OSCC has been explored with a special focus on how oral microbiota induces OSCC initiation and stemness by modulating the oral mucosal stem cell and CSC niche defense.

## Introduction

Oral cancer is one of the common head and neck cancer (HNC) type in the world and 90% of these are oral squamous cell carcinoma (OSCC) (1). According to Globocan, 3,77,713 cases of oral cavity and lip cancer were diagnosed in 2020, with 1,77,757 deaths reported (2). Early detection being a challenge in oral cancer, are usually diagnosed during stage III or IV disease state (3). Specifically in developing country like India, 60-80% of oral cancer patients are presented at advanced stage of the disease in comparison to 40% in developed countries (4, 5). Surgery followed by chemo/radiation is the mode of treatment, whereas, platinum monotherapy is the standard of management in stage IIIb and stage IV OSCC (6, 7). However, nearly 60% people show resistance to platinum therapy (8). Despite advances in treatment, the overall five year survival rate in OSCC patients is 50% across the globe (9).

During the last three decades tremendous progress has been made in understanding the complexity of OSCC. Oral leukoplakia (OLK) is the most common pre-cancerous lesions of oral epithelium and about 10-15% of OLK may transform into malignant growth (10). In oral cancer, multiple genetic events alter the function of oncogenes such as RAS and tumor suppressor gene p53 (11). These oncogenic events alter the normal function of growth factors, including TGF-α and EGF during early-stage oral carcinogenesis (12, 13). Tumor suppressor gene p53 is mutated in the majority of head and neck cancer (13). Clinically, p53 mutations correlate with poor prognosis and worse patient survival. Mutations in p53 either hinders its direct binding to the p53-responsive element in DNA (e.g., p53R273H, p53R280K), or alters the protein conformation to disrupt its functionality (14). Additionally, changes in epigenetic levels in p14/ARF promoters, methylation of the p53 promoter or persistent expression of MDM2 and MDMX may also contribute to loss of function of wild type p53 (15). In the OLK lesions, oncogenic events, including p53 mutation have been reported (16, 17). However, these oncogenic events may not be enough for malignant transformation, as only 10-15% of OLK transform to malignancy (10). Pro-inflammatory cytokines such as IL-1α, IL-6, and TNF-α may be involved in promoting OLK carcinogenesis (18).

Once the pre-cancerous lesion progresses to malignant growth, the tumor microenvironment (TME) undergoes profound changes. The TME comprises of cancer cells, endothelial cells, stromal cells, cancer stem cells (CSCs), and immune cells (19). In the TME of OSCC, CSCs interact with stromal cells and other immune cells to induce tumor progression and invasion (20). Chronic inflammation and associated inflammatory pathways such as toll-like receptor (TLR) pathways, and NFκB transcription factor play important role in modulating the TME (21). Such inflammatory and oxidative stress microenvironment may promote cancer stemness in CSCs as well as in non CSCs. Notably, studies showed TLR plays an important role in inflammation induced cancer stemness (21–23). Activation of TLR2/TLR4 aids in tumor progression by inducing the expression of genes associated with stemness i.e OCT 4, NANOG and SOX 2 in cancer cells (23). These stemness associated transcription factors are expressed by CSCs (22). CSCs are the most aggressive cancer cells having self-renewal and migratory capacity (24, 25). CSCs of OSCC have been identified and may play a role in relapse (26, 27). Different pathways including NOTCH (28), Wnt/β-catenin (29), Sonic Hedgehog pathways (30) and HIF-2α/MYC stemness pathway (31) were shown to maintain CSC stemness. CSCs reside in their niches, which are often hypoxic (32). CSCs may defend their niches from tumor immune response, oxidative stress as well as therapy induced stress by reprogramming into a highly invasive, inflammatory and immunosuppressive phenotype called the tumor stemness defense (TSD) phenotype (23, 32). MYC-HIF-2α stemness pathway may be involved in reprogramming of CSCs TSD phenotype (23, 32).

Oral microbiota that include bacteria, virus and fungi are present in the oral cavity may directly or indirectly promote OSCC initiation and progression by increasing inflammation and oxidative stress (33–35). Certain oral bacteria may play a proactive role in the initiation and progression of OSCC as these pathogens are found to be disproportionately present in cancerous tissues (36). Numerous studies have shown that enhanced growth of pathogenic oral microbiota contributes actively to carcinogenesis (33, 35, 37) as well as tumor progression of OSCC (34) by enabling a pro-tumorigenic and chronic inflammatory TME (35, 36, 38–40). However, how oral microbiota interact with the components of TME and maintain stemness in cancer cells for tumor progression has not been discussed extensively.

**Figure 1:**
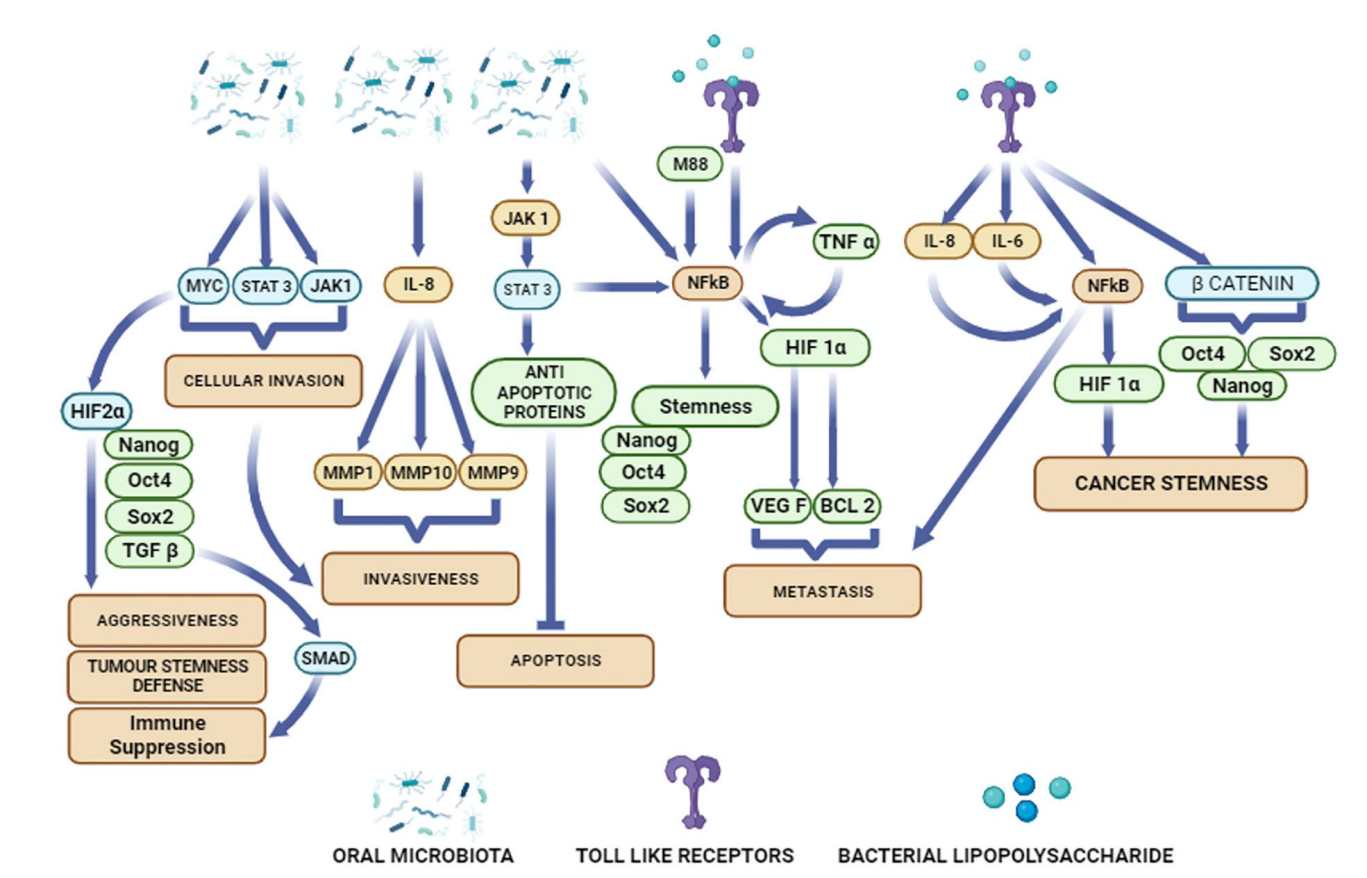
Potential activation of diverse cellular pathways in oral cancer cells by oral microbiota and bacterial metabolites.

In the era of targeted therapies and immunotherapies, it is important to gain new insight on oral cancer microbiome, and their putative role in OSCC growth and therapy resistance. The objective of this review is to discuss oral microbiota’s role in carcinogenesis, tumor progression and cancer stemness in OSCC. To assess oral microbiota’s role in OSCC initiation, progression and stemness, we have focused on microbiota that can activate inflammatory pathways (**Figure 1**). We then examined the role of these inflammatory pathways in modulating cancer stemness as well as CSC niche defense.

## Microbiota’s role in OSCC initiation/progression

Oral microbiota comprises a heterogeneous microbial population, inhabiting in different parts of the oral cavity such as teeth, gingiva, and tongue (34). The microorganisms of oral cavity may be present as commensals, symbionts and pathogens (41–43). These microorganisms maintain a balanced state with the host that benefit each other. However, during disease state, such as periodontitis, increased growth of a few commensal microbiota with concomitant declination of other oral microbiota in the oral cavity may occur (44, 45). This alteration in the microbial population is inferred as dysbiosis (46). Dysbiosis may increase the risk of OLK. In a population based study, Meisel et al. reported that severe periodontitis increased the risk of leukoplakia (47). Although the mechanisms is not yet clear, periodontitis and associated dysbiosis of oral microbiota can lead to excessive inflammation which may create anaerobic environment enriched with tissue breakdown components (48). This further allows growth of proteolytic gram negative bacteria which uses essential amino acids and hemin as an energy source (48). Pathogens such as PG and *T. forsythia* were found to over express potential virulence genes such as various Ton-B receptors, peptidases, proteases, hemolysins and other genes responsible for bacterial aerotolerance, iron-transport (49). Notably, Yost et al. also reported enhanced activity of FN up regulates putative virulence genes which are related to proteolysis, sodium-ion transport, and cellular response to phosphate starvation leading to dysbiosis (49). Thus, chronic inflammation and dysbiosis may favor the evolution of certain bacterial species contributing to the genesis of OLK. Indeed, OLK lesions are found to be enriched with specific bacterial species that include *Fusobacterium*, *Leptotrichia*, *Campylobacter* and *Rothia* species, with *Fusobacterium* being highly prevalent (50).

Microbiota present in OLK lesions may secrete onco metabolites that contribute in the carcinogenesis process. Recently, Amer et al. reported that residence of *Rothia mucilaginosa* in OLK may lead to escalated production of acetaldehyde (ACH) in the oral cavity (51). ACH induces oxidative stress in oral keratinocytes (51) which causes functional and structural changes in the DNA that effects cell cycle and thereby play a role in initiation of carcinogenesis (52). Importantly, oral bacterial species such as *Streptococcus salivarius*, *S mitis*, *S bovis*, *Veionella spp*, *Staphyloccus aureuss*, *Epidermis nocordia spp* in the oral cavity can convert nitrate into nitrite (53), which are carcinogenic metabolites associated with oral carcinogenesis (54).

### Virus and fungi in OSCC development

Among the viruses, HPV has been reported to be associated with OSCC by many researchers (55–59). The HPV-16 integrates its genome in specific sites of HNSCC tumor. The integrated genome consists of seven early genes namely (E1-E7) and two late genes (L1-L2). E6 protein is involved in degradation of p53 while E7 protein induces cell cycle to go beyond restriction point (G1-S checkpoint) into S phase. Increased survival as well as accumulations of DNA damage and mutations over cell replications results in malignant transformation and development of carcinomas (59). The Epstein-Barr virus (EBV) was found to be involved in nasopharyngeal cancer (60, 61), OSCC and potentially malignant oral diseases including gingivitis, and periodontitis. This virus can establish latent infection in the B-lymphocytes and salivary gland cells (61). Using FaDu oral cancer cell line, researcher reported that Coinfection of HPV and EBV can induce the tumorigenicity of oral cancer (62). Many research group has been studying the association of herpes simplex virus (HSV), an adenovirus with oral cancer in clinical subjects and animal model (63, 64). A recent study has shown that HSV-1 is predominant in oral cavity and tumor tissue of OSCC subjects, however has no significance in OSCC cell survival and invasion (65). A population-based study showed hepatitis C virus (HCV), not hepatitis B virus (HBV) as risk factor for oral cancer (66). In addition to virus, fungal microorganism such as *Candida* has been identified in OLK lesions (50, 67). Study reported the high abundance of *Candida* (78.8% of all OSCC cases) and Saccharomyces (76.8% of all OSCC) in OSCC patients versus healthy control (68). Importantly, dysbiosis of oral bacteria due to the use of broad spectrum antibiotics may pave the way for the growth of hyperplastic Candidiasis that may contribute to OLK genesis (69, 70) as well as OSCC (71). *Candida* infection leads to induction of cytokines such as IL8 and TNF-α to activate TLRs which can interact with NF-kB inflammatory pathway in metastasis of oral cancer (72). Thus, dysbiosis creates a chronic inflammatory state, where bacteria, fungal metabolites, as well as the pro-inflammatory cytokines may lead to the genesis of OLK, and then subsequent malignant transformation.

### Oral microbiota for cancer staging

Studies have shown that cancer staging could be achieved by the presence of specific microbiota, indicating the association of specific bacterial population in each stage of malignant progression (37, 73). Indeed a study by Yang et al. reported that the relative percentage of bacteria in saliva increased from stage 1 to stage 4 of OSCC patients as compared to healthy controls. The *F. periodonticum* percentage was found to be increased in oral saliva of OSCC patients from 1.66% in stage 1 to 2.41% in stages 2 and 3, further to 3.31% in stage 4. Some other bacteria including *Parvimonas micra, Streptococcus constellatus*, *Haemophillus influenza*, *Filifactor alocis* were also found to be increased in the same patients from stage 1 to stage 4 (73). Depending on the precancerous or OSCC stage, bacteria including *Fusobacterium* spp., *Prevotella* spp., *Haemophilus* spp., and *Campylobacter* spp. found in the samples express specific associated functional pattern (37).

### Healthy commensal oral microbiota against cancer

Importantly, many commensal microbiota in oral cavity are reported to be healthy in context of carcinogenesis or OSCC progression (73–75). Researchers found *P. Pasteri*, a commensal bacterium present in oral saliva of healthy individual decreases in oral saliva of OSCC patients (73). This research group further found *Streptococcus mitis* and *Haemophilus parainfluenza* as prominent bacterial species associated with healthy individuals (73). Using OSCC cell lines, another group of researchers reported that *S mitis* bacterial lysate can inhibit OSCC cell proliferation (76). Another study observed a decrease of the presence of *Actinobacteria*, in tumor samples (74, 77). *Actinomyces* spp., known for its ability to safeguard the mucosa by releasing protease inhibitors that hinder tumorigenesis, becomes outnumbered due to the acidic TME and hypoxia (74). Furthermore, high relative abundance of the fungi *Malasezzia*, were shown to favor survival of OSCC patients (68). These studies indicate that many commensal oral microbiotas of oral cavity have the ability to reduce cancer progression.

### Sequencing based analysis of the microbiota associated with OSCC

The sequencing based analysis may provide more insight into the association of microbiota in different cancer including OSCC (49, 76, 78, 79). Indeed in a sequencing analysis in clinical subjects, Yost et al. showed a significant alteration in the metabolic properties of certain anaerobic bacteria in the development of periodontitis induced dysbiosis (49). Application of next generation sequencing (NGS) technologies such as 16S rRNA sequencing has provided data on oral microbiota dysbiosis in oral cancer (74, 80). The 16s rRNA gene amplicon sequencing techniques has provided greater insight on dysbiosis in various oral cavity diseases (78) including periodontal diseases, OLK, dental caries, gingivitis and OSCC (81). Hooper et al. found heterogeneous viable bacterial population in the cancer cells and TME of primary OSCC using 16S rRNA sequencing (78, 82). Another 16s rRNA sequencing study revealed the presence of saccharolytic and acid-tolerant bacterial populations such as *Prevotella melaninogenica*, *Staphylococcus aureus*, *Veillonella parvula, Micrococcus* inside the tissues derived from tumorous specimens. Concurrently, in the adjacent non-tumor tissues, they found the presence of *Moraxella osloensis*, *Prevotella veroralis*, and *Actinomyces spp* (78). Furthermore, an interesting case control study by Perera et al, evaluated the potential role of the presence of microorganism in deep tumor tissue samples of OSCC patients. The cohort consisted of 25 OSCC cases and 27 fibro epithelial polyp (FEP, FEP is common reactive hyperplasia of oral mucosa) cases as controls from Sri-lanka. The 16S rRNA gene sequencing of V1-V3 region from tissue samples reported the presence of microorganisms such as *Fusobacterium nucleatum subsp polymorphum*, *Pseudomoas aureginosa*, and *Campylobacter concisus* in OSCC cases. The presence of *Streptococcus mitis*, *Rothia* spp. and *Lautropia mirabilis* was observed in the FEP cases. These bacteria showed pathways such as isoleucine biosynthesis, glycolysis/glucogenesis as well as base excision repair pathways (83). The functional prediction analysis in the OSCC cases reported inflammatory molecule lipopolysaccharides (LPS; bacterial endotoxin secreted by anaerobic bacteria) biosynthesis pathway and energy metabolism pathways. A single cell-based RNA sequencing method named INVADE seq used to study the microbial populations in OSCC clinical subjects (n=7) reported intratumoral microbial heterogeneity (84). Bacterial species were found to reside in specific intratumoral niche as well as *Fusobacterium* and *Treponema* sp. were found to modulate OSCC progression (84). A machine learning based study reported the presence of bacteria genera *Prevotella*, *Stomatobaculum*, *Bifidobacterium* in OSCC clinical subjects (n=54) which positively correlated with lymph node mediated metastasis aiding in OSCC (85). Furthermore, in clinical subjects (n=41), using a bioinformatic approach Arthur et al. reported that the OSCC associated microbiome comprised of *Fusobacterium* spp., *Prevotella* spp., *Haemophilus* spp., and *Campylobacter* spp. were in different proportion at different stages of malignancy than in control subjects (37). These sequencing data provides us with new opportunities to study the host-pathogen interaction between microbiota and oral cancer cells.

### Molecular mechanisms of microbiota induced OSCC initiation/progression

The molecular mechanism of bacterial induced OSCC initiation and progression may be due to the inflammation medicated host defense response. Using *in-vitro* assay of specific bacteria and OSCC cell lines, specific oral bacteria populations such as PG and FN have been identified to induce inflammatory cytokines along with cellular invasion of OSCC (86). Ha et al. reported that invasive ability of PG bacteria during chronic infection of Ca9-22 OSCC cells was mediated by cytokine IL-8 (77). Chronic infection of the OSCC cells by the PG bacteria up-regulated the expression matrixmetalloproteins (MMPs) such as MMP 1 and MMP 10 via IL-8. Importantly when the cytokine IL-8 was inhibited by specific siRNA, the expression of the MMPs were down-regulated and the invasive ability of the OSCC cells was also reduced (77). In another *in-vitro* study, Harrandah et al. reported increased secretion of cytokine IL-8, MMP-1, and MMP 9 in OQ01 cells (primary head and neck cancer cell line) infected by live FN bacteria. The increased secretion of IL-8 promoted cancer cell invasion. Interestingly, the secretion of IL-8 was mediated by LPS as inhibition of LPS by polymyxin B treatment reduced the secretion of IL-8 in the bacterial culture supernatant (**Figure 1**) (40). More *in-vitro* studies using bacterial populations isolated from OSCC lesions, as well as the use of primary OSCC cells may provide valuable information about the host-pathogen interaction between oral bacteria and OSCCs. Importantly, such studies need to investigate the potential internalization of bacteria into the cancer cells, as clinical studies done in breast and colorectal cancer showed the presence of intracellular bacteria in cancer cells obtained from primary tumors (87). The presence of intracellular bacteria in OSCC has not yet been studied well. Using an *in vitro* model of the SCC-25 cancer cell line, we showed that FN present in the saliva of oral cancer subjects internalize to the ABCG2+/EPCAM+ population of SCC-25 cells (88, 89). We also recovered FN from the primary ABCG2+/EPCAM+ population of relapsed OSCC (90). However, it is not clear how a few intracellular bacteria may induce stemness and OSCC progression.

### Experimental models of microbiota induced OSCC development

Bacterial secretory products may activate inflammatory pathways such as TLRs, which may contribute to chronic inflammatory state of TME. Lipopolysaccharide (LPS), an endotoxin, is a key bacterial product that is secreted from the outer membrane of gram-negative bacteria (91). The LPS of oral bacteria FN-activated inflammatory cytokines IL-6, and TNF-α may affect development of cancer by influencing apoptotic pathways. Moreover, the LPS of oral bacteria PG can elicit TLR 4 response that prevents apoptosis and promote tumor cell proliferation as well as invasiveness (**Table 1**) (98). Activation of TLRs in turn can activate NF-κB signalling in the TME of oral cancer and sustain a chronic inflammatory state (93), which may promote cancer progression. However, TLRs are also known as double edged sword in either favoring cancer progression (99), or inhibit cancer progression (100, 101). TLRs present in oral mucosa (102) can recognize the pathogenic microbiota and can trigger an immune response through the direct recognition of ligands derived from the microbiota (103, 104). Therefore, TLRs may recognize the pathogenic microbiota that induces cancer progression and activate an anti-tumor effect via eliminating the microbiota by triggering immune response. In an *in vitro* setting, *S mitis* bacterial lysate was reported to inhibit proliferation of three OSCC cell lines; Cal 27, SCC 25 and SCC 9 by up regulating the expression of the pro inflammatory cytokines IL 6 and TNF-α (76). These studies indicated that the up regulation of the pro inflammatory cytokines also have anti-cancer effect in OSCC.

**Table 1:** List bacteria and virus associated with cancer and the expression of stemness gene markers or stem cell surface markers.

Presence of bacteria and virus	Cancer Type	Bacterial products/Viral Proteins	Expression of stemness gene marker/Stem cell marker	Reference
**Human papilloma virus (HPV)**	**Oral Squamous cell Carcinoma**	**E6**	**CD44,HIF-2αNANOG,SOX 2,OCT 4**	(89)
**Hepatitis B virus**	**Colorectal cancer**	**X antigen**	**OCT-4, NANOG, β catenin, KLF 4, and EpCAM**	(92)
***Porphyromonas gingivalis***	**Oral Squuamous cell Carcinoma**	**Lipopolysaccharide**	**CD44, CD 133**	(93)
***Fusobacterium nucleatum***	**Colorectal cancer,**	**FAD A**	**NANOG, OCT 4, SOX 2**	(94)
***Fusobacterium nucleatum***	**Oral Squamous cell Carcinoma**	**FAD A, FAP 2**	**NANOG, OCT 4, SOX 2**	(95)
***Bacteroides fragilis***	**Colorectal cancer**	**Lipopolysaccharide**	**SOX 2, NANOG**	(96)
***Trepenoma denticola***	**Oral squamous cell carcinom**	**Lipooligosaccharide (LOS)**	**Integrin α V**	(97)

Bacteria and Viruses are associated with different cancer and these microorganisms induces expression of varied stemness gene marker.

To further evaluate the role of microbiota in OSCC initiation and progression, mouse models are required. Among several mouse models of OSCC, 4 NQO (4-Nitroquinoline 1-oxide) induced carcinogenesis mouse model is well characterized (95, 105). In this 4NQO mouse model, Gallimidi et al. demonstrated that co-infection by PG/FN promoted tumourigenesis through inflammatory response of TLRs (86). The new murine model comprising of FN and PG induced chronic periodontitis were subjected to 4-NQO exposure. It was found that the presence of the FN and PG microorganisms significantly up regulated the IL6-STAT-3 signaling axis, aggravating the OSCC progression. To corroborate the *in-vivo* study results, they carried out an *in-vitro* study using an assay of FN and PG co-infection of OSCC cells and found the enhanced activation of TLRs leading to high expression of IL-6. Importantly, the co-infection significantly changes the proliferation rate and characteristics of the OSCC cells (86). In another *in-vivo* study of 4-NQO induced carcinogenesis, transfer of oral bacteria from tumor bearing mice into germ free recipients significantly increased the numbers and sizes of tumors (46). While these mouse models are important to understand the complexity of bacterial and host cell interaction leading to carcinogenesis and tumor progression, their clinical relevance for human cancer is not yet clear. Future studies need to be conducted using humanized mouse model of oral cancer.

## Oral microbiota induction of tumor stemness

The above discussions make it clear that the oral microbiota contribute in tumor progression due to chronic inflammation and modulation of the TME. However, how the microbiota modulate the TME need serious attention. The microbiota may interact with the oral mucosa cells and CSCs in the TME to induce stemness for tumor initiation and progression (95, 106).

### Stemness and cancer stemness

Stemness is the key attribute of stem cells characterized by self-renewal and undifferentiated state (32, 107). It is an integrated molecular program comprising of signalling pathways, epigenetic and gene expression networks that sustains the self-renewal and undifferentiated state of stem cells (32, 107). Epigenetic mechanisms such as DNA methylation, histone modifications and non-coding RNA play a key role in the stemness of both normal and cancer stem cells (108, 109). These epigenetic mechanisms regulate transcription factors involved in stemness. The OCT 4, NANOG, SOX 2 are the transcription factors or genes that maintain stemness in embryonic stem cell (110) and may play a key role in cancer stemness too (22). Stemness in differentiated cells can also be induced by these transcription factors in combination with MYC, and KLF4 (110). Interestingly, stemness may be induced in differentiated cells and also in non CSCs by up-regulation of the stemness transcription factors (111). In hematopoietic stem cells (HSCs), stemness is regulated by development pathways such as Wnt and NOTCH pathways, as well as VEGF/HIF-1 autocrine pathways (112). These pathways regulate the integrated gene expression networks that maintain the stemness attractor state in the epigenetic landscape (112). Epithelial cells may enhance stemness to undergo epithelial mesenchymal transition (EMT), an important mechanism in developmental biology. In the EMT process, transcription factors play key roles, and these transcription factors may also contribute to the balance between stemness and differentiation (32).

Cancer stemness has been a key feature of CSC self-renewal to sustain tumor growth and progression (113). We have shown that upregulation of stemness genes in CSCs are maintained by a MYC-dependent HIF 2α stemness pathway (**Figure 1**) that transiently suppresses the expression of p53 tumor suppressor gene (31). Inflammatory mediators are also reported to up regulate stemness genes in several cancer types (114–116). Toll-like receptors (TLRs) and nuclear factor-κB (NF-κB) (**Figure 1**) are two key inflammatory mediators that contribute in cancer initiation and progression. Importantly, TLR3 and TLR4 are highly expressed in OSCC patients (21). Activation of these TLRs stimulates the expression of HIF1-α via NF-κB and thus, maintain the cancer stemness in OSCCs (**Figure 1**) (21). In breast cancer cells, TLR3 trigger the co-activation of β catenin and NF-κB, which further enhances the expression of OCT3/4, NANOG, and SOX2 stemness genes (115). In hepatocellular carcinoma derived CSCs, LPS based inflammatory mediators were shown to enhance the expression of stemness genes OCT4 and NANOG via IGF-IR signalling pathway (114). CSCs enhance proliferation of the non CSCs in the tumor niche by inducing the expression of the multipotent stemness genes (117). Recently, Shin et al. reported that enhanced expression of OCT 4 in CSCs increases the tumorigenic potential of OSCC (25).EMT, the process of conversion of epithelial cells into mesenchymal phenotype may also induce cancer stemness (118). Epigenetic mechanisms are also involved in stemness and cancer stemness. A study reported that methylation of DNA in the NANOG promoter may induce a switch of non-CSCs to CSCs (119). Inflammatory pathways may promote abnormal DNA methylation leading to carcinogenesis and tumor progression (108, 109). Several histone modification enzymes such as KDM5B and KDM1A are key regulators of stemness and cancer stemness (120). Non-coding RNAs are also involved in phenotypic plasticity and cancer stemness (121). The role of these epigenetic modifiers in the head and cancer has been studied (122). Particularly, p16INK4a is the most hypermethylated gene in oral cancer (123), and this gene maybe a target of inflammation-induced epigenetic modification in cancer, as shown recently (108).

### Microbiota and cancer stemness

Many studies have shown the role of microbiota in the stemness of various cancer types as outlined in the **Table 1**. However, only a few studies are reported on OSCC. A positive correlation was reported about the presence of *Bacteroides fragilis* and FN level with the expression of OCT-4, NANOG and SOX2 in colorectal cancer patients (94, 96) (**Table 1**). Microbiota may also induce stemness by regulating the mechanisms of EMT. A study has shown that prolonged and repetitive infection with PG promotes EMT-like changes and stemness in OSCC cells (77). Another study, demonstrated that infection by FN induced high expression of oncogenes STAT 3, MYC, JAK 1 in OQ01 cells (primary head and neck cancer cell line) (40). HPV+ve oropharyngeal cancers (OPSCCs) is enriched in CSCs (124–127). Expression of CSC markers CD44 (128) and ALDH1 (126) were found to be higher in the HPV positive oral cancer patients compared to HPV negative oral cancer patients (126, 128). Hepatitis B virus (HBV) encoded X antigen (HBVx) was reported to activate stemness associated factors OCT-4, NANOG, β-catenin, KLF 4, and EpCAM as well as induce cell migration and sphere formation in hepatocellular carcinomas (HCC) (92).

The fungi has both cancer development and anti-cancer properties (129). Candida has been extensively reported for cancer progression (130), whereas, *Ganoderma lucidum* polysaccharides reduce cancer stemness by inhibiting EMT (131). These studies indicate that bacteria, virus as well as fungi may induce cancer stemness in OSCC.

### Molecular mechanism of microbiota-induced cancer stemness

The molecular mechanisms of microbiota-induced cancer stemness are not yet clear. Cancer stemness may be induced by bacterial metabolites such as LPS and Lipooligosaccharide (LOS) (91, 97). Oral bacterium, *Trepenoma denticola* has been reported to induce stemness, by enhancing cellular migration and tumorosphere formation. This contributes to an aggressive OSCC phenotype via LOS mediated TLR4/MyD88 and integrin/FAK signalling pathways (**Table 1**) (97). LPS is known to induce immune response, and many studies have shown that some bacteria, such as *P. multocida* are dependent on LPS for infection (132). Several inflammatory cytokines IL1β and TNFα were reported to be stimulated by LPS of oral microbiota (133). Interestingly, different inflammatory cytokines were reported to upregulate the MMPs production which triggers cancer cell invasion (134). LPS meditated TLR 4 expression induces EMT (135) and stemness (90) thereby aiding in tumour progression and metastasis (135).

These findings indicate that oral bacteria secreting LPS may induce stemness in oral cancer cells mainly by activating inflammatory pathways. Importantly Ha et al. showed that PG invaded OSCC cells led to increased secretion of IL-8 and VEGF as well as increased stemness and expression of CD44 (77) (**Table 1**). Microbe induced TLR expression in oral cancer cells may increase the transcription of NF-κB that in turn promote cancer stemness (**Figure 1**). HPV was reported to promote tumor growth by enhancing cancer stemness via miR-181a/d regulation in OPSCC cells (136). Thus, oral microbiota may employ various mechanisms to modulate stemness in the oral cancer cells.

## Microbiota interaction with stem cell and CSC niche defense

The molecular and cellular mechanisms by which microbes induce cancer stemness are not fully understood. One of the possible mechanisms is that microbes may promote inflammation and oxidative stress in the CSC niche, which can then activate the CSC niche defense and associated signaling pathways involved in stem cell self-renewal, and niche modulatory stemness (112).

To understand more about the host-pathogen interaction between microbes and CSCs in the niches, we need to learn more about the stem cell niche defense mechanism including the oral mucosa stem cell niche (137). Studies have shown that stem cell niche cells such as stromal cells immune cells and the extracellular matrix defend the niche against internal as well as external stresses in order to maintain the stem cell pool. The stemness is either niche dependent i.e. stem cells are regulated by paracrine signaling of the niche cells or niche independent, i.e. stem cells can maintain stemness in a cell-intrinsic manner (112). Several signaling pathways such as Wnt, NOTCH, TGF-beta, NFκB, VEGF/HIFs autocrine signaling pathway, and PI3/Akt/mTOR pathways were shown to be involved in maintaining their niche integrity by promoting cell-cell communication, metabolic regulation, immune surveillance, extracellular matrix remodeling and regulating inflammation, especially in the bone marrow stem cell niche (112). In addition to these mechanisms, stem cells may defend their niches by reprogramming to enhanced stemness states that exhibit altruistic behavior (112, 138). We have developed *in vitro* and *in vivo* models to identify this stem cell-specific innate defense mechanism in the hESCs (110), MSCs (112, 138–140), and oral mucosa stem cells (89, 141). We found that during stress hESCs and MSCs reprogram to a robust state of cytoprotection, the altruistic stem cells (ASCs) state by up regulating HIF-2α stemness pathway. ASCs secrete antioxidants, growth factors as well as anti-pathogen agents to protect the stem cell pool residing in the niche (110). Importantly, in a model of murine hepatitis virus-1 (MHV-1)/Mycobacterium tuberculosis/MSC host-pathogen interaction (140), the infected MSCs of alveolar niche exhibited ASC mediated defense against viral infection. This unique stem cell niche defense may also make stem cells pro-oncogenic.

### Stem cell niche defense in cancer initiation

Microbial interaction with stem cell niche defense may promote carcinogenesis. The ASC phenotype exhibits transient suppression of the p53 tumor suppression gene, but exhibits very high transcriptional activities of NANOG, SOX2 and OCT4. The high expression of these factors may provide a self-sufficiency state by yet unknown mechanisms (110). Therefore, the ASC phenotype could be the target for malignant transformation. Interestingly, in a 4-NQO model of oral mucosa stem cell reprogramming to ASCs (**Table 1**) (141), the addition of FN or HPV-16 markedly increased the malignant transformation of oral mucosa stem cell-derived ASCs to CSCs (141). Notably, we identified FN together with HPV 16 has the ability to reprogram human primary CD271+ oral keratinocyte progenitor into ASC state (89) (**Table 1**). Thus, the microbial interaction with the ASC mediated stem cell niche defense may play important role in the progression of pre-cancerous lesions to malignant growth.

### Stem cell niche defense in cancer progression

**Figure 2:**
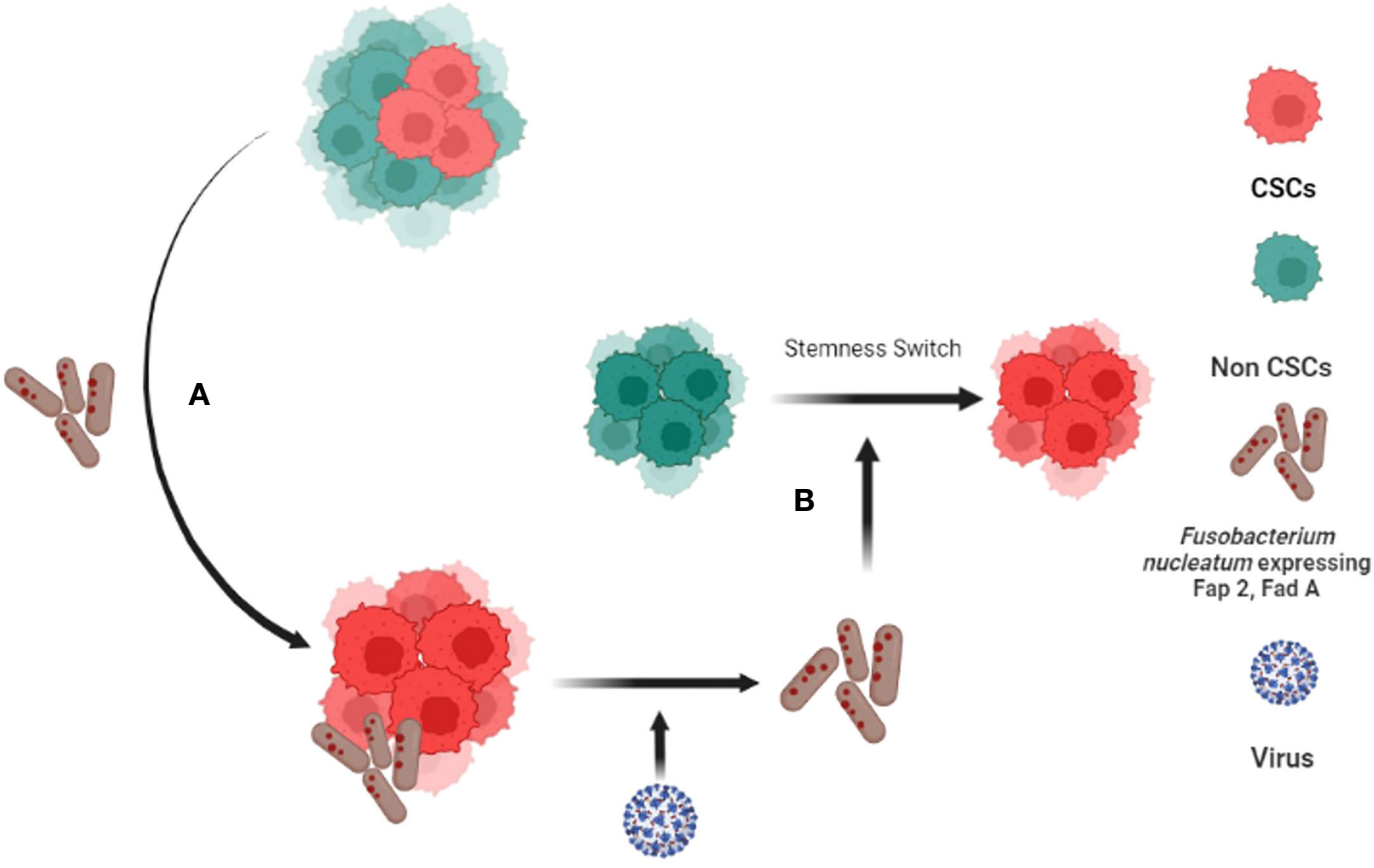
*Fusobacterium nucleatum* (FN) induced tumor stemness switch of Non-CSCs to CSC. **(A)** CSCs interact with *F. nucleatum*and reprogram to tumor stemness defense (TSD) phenotype. The TSD phenotype enhances the expression of Fap2 and Fad A in FN. **(B)** The reprogrammed FN along with HPV-16 then reprograms Non Stem Cancer Cells (non-CSCs) to CSCS by inducing tumor stemness switch (88, 89).

This unique altruistic defense mechanism of stem cell niche may be hijacked by CSCs to defend their niche (138). CSCs may reprogram MSCs to ASC phenotype to modulate a cytoprotective and immunosuppressive TME (142). CSCs may make transition to a higher state of stemness to defend the niche against stress such as oxidative stress, chemotherapy, as well as pathogen invasion (138). We showed that CSCs might reprogram to a transient but highly inflammatory stemness state when exposed to extreme hypoxia/oxidative stress (22), and chemotherapy (111); the phenotype could protect CSCs and non-CSCs in the niche from oxidative stress (111) as well as pathogen invasion (23). Therefore, we termed the phenotype as tumor stemness defense (TSD) phenotype (23). Our ongoing work suggests that the TSD phenotype can be isolated from primary OSCCs obtained from stage III and stage IV locally advanced cases (23) suggesting potential clinical implications of the TSD phenotype. Moreover, we found that while FN can induce TSD phenotype in SCC-25 derived CSCs and transform non CSCs to CSCs (**Figure 2**) (88, 95), *Myocobacteria* can induce apoptosis of the TSD phenotype (23). Thus, bacteria may either exert pro or anti-tumor effects based on their effect on the CSC niche defense. Interestingly, a retrospective analysis of OSCC subjects revealed less frequent relapse in the FN positive subjects possibly due to enhanced immunogenicity by FN (143). Future studies are required to investigate the microbes/CSC niche defense interaction to understand microbe’s contribution in cancer stemness or cancer reduction. Potential future studies include microbial interaction with stemness pathways that switch non-CSCs to CSCs (117), epigenetic pathways that promote stemness (144), MYC-HIF-2α stemness pathways that reprogram CSC to TSD phenotype (22, 88) and embryonic stemness related transcription factors that promote OSCC progression (25). In this context, we have developed an *in vitro* model of FN and OSCC derived CSC host/pathogen interaction to study the microbiota-induced TSD phenotype (**Figure 2**).

### Triangle of evils: microbiota, CSC niche defense and immunosuppressive TME

Following the rapid approval of a few immune check points’ inhibitors (ICI) as immunotherapy agents for melanoma, lung cancer and renal cancer, there is a growing interest to study the mechanisms of immunosuppression in other tumor types, where ICI has failed to show satisfactory clinical response. ICI based immunotherapy effectiveness in head and neck squamous cell carcinoma is very poor due to immunosuppressive TME that hinders T cell infiltration (145). Here we shall discuss the possible ways that the stem cell niche defense and microbes contribute to immunosuppressive TME in OSCC. A growing body of research suggests the pathological communication between CSCs and immune cells that shape the immunosuppressive TME in diverse tumor types (146–149). Briefly CSCs are intrinsically immunosuppressive (149, 150) and reside in the immunopriviledge niche of hypoxia (32). Several stemness pathways active in CSCs such as TGFβ, Wnt/β-catenin and NOTCH pathways inform immune cells for immune evasion (146–149, 151). In this context, only a few studies have been done on the immunosuppressive properties of HNSCC and OSCC derived CSCs. One study found that CD44+ cells of OSCC cross-talk with macrophages to promote stemness activities (152) while another study reported STAT3 mediated expression of PDL-1 expression in CD44+ CSC leading to suppression of T cell mediated tumor immune response in OSCC. We found that OSCC derived TSD phenotype of CSCs shape immunosuppressive TME by secreting immunosuppressive cytokines and also reprogramming mesenchymal stem cells and innate immune cells (142). Although the physiological basis of CSC-immune cell crosstalk is not yet clear it is possible that CSCs hijack the reciprocal communication between adult stem cell and immune cell in the stem cell niche. Both hematopoietic and mesenchymal stem cells interact with the immune cells to maintain stem cell homeostasis (153). The crosstalk involves stemness pathways and therefore it is not surprising that the CSC niche defense hijacks the stemness pathways such as TGF β and Wnt- β-catenin to evade tumor immune response. However, it is not yet clear if and how microbes define CSC and immune cell crosstalk. CSC may adopt intrinsic mechanisms of innate immune cells to defend the niche against microbial invasion. We demonstrated that *M tuberculosis* and BCG infected CSC activate the innate immune defense of bystander apoptosis to eliminate intracellular microbes. This mechanism of CSC niche defense can be exploited to eliminate the TSD phenotype of CSC obtained from diverse tumors (23). CSC may also reprogram microbes to enhance tumor stemness and immunosuppressive TME (**Figure 2**). These reprogrammed intracellular microbes may then modulate crosstalk between CSC and immune cells and aid in resistance against immunotherapy as well as chemotherapies (106). Indeed, cisplatin treated oral cancer CSCs (23) exhibit NK cell suppressive activity in the presence of *Fusobacterium nucleatum* (89). Then chemotherapy may also modify the crosstalk among the microbes, CSC, and immune cells in the niche. Understanding the molecular and cellular mechanism of the crosstalk between CSC niche defense and microbes may better inform the reciprocal communication between CSCs and immune cells and consequently develop new therapeutic avenues.

## Future research on microbiota and cancer stemness

The host-pathogen interaction among stem cells niche, microbes, cancer cells and the immune cell components is complex, which may alter immune response contributing to cancer initiation and progression. Novel organoid models as well as single cell transcriptomics studies may provide experimental basis to gain insight about the complex interaction, and thus help us elucidate the immunosuppressive component of the stem niche defense. Such organoid systems may also be exploited to study the microbial interaction with the stem cell niche defense i.e study the potential gain of extreme degree of self-sufficiency by the cancer cells to transform their niches in order to resist the entry of other microbes (140). Another experimental model to consider is the germ-free mouse model of carcinogenesis, combined with *in vitro* model of stem cell and microbial interaction. We and colleagues showed that microbial colonies obtained from actively growing mouse oral cancer lesion of syngenic mice enhances 4NQO carcinogen-induced malignant transformation of oral epithelial cells (46). Subsequently, using an *in vitro* model of stem cell and microbial interaction, we found that HPV-16 and FN induces stem cell altruism in the CD271+ oral mucosa stem cells, and these ASCs then undergo malignant transformation following exposure to the 4NQO carcinogen (89, 141). Further development of these models and also exploiting stem cells/microbial interaction models (154) may help us to explore the biology of oral mucosa stem cell/oral microbiota interaction and its consequence on stem cell niche defense that may lead to immunosuppression in the niche leading to promotion of carcinogenesis. Identification of stemness pathways of the interaction between stem cell niche, microbe and cancer could lead to identify attractive immunotherapy targets against CSCs.

Another avenue of future research is to develop organoid model to study the immunomodulation of stem cell niche as well as CSC niche by the host-microbe interaction. The complex interaction among dysbiotic microbiota, stem cell niche, TME constituents such as immune cells, MSCs and CSCs may reprogram the TME into a pathogen-favoring microenvironment. Specifically, such complex evolution of microbe’s interaction may lead to the stem cell niche defense which may contribute to CSC dormancy and relapse. To explore such possibilities, it is important to develop organoid as well as organ on chip models and explore the single cell transcriptomics to delineate the architecture of host pathogen interaction.

Importantly, these *in vitro* and *in vivo* models may be utilized to develop novel therapy to target the microbial induction of oral cancer stemness. Studies have shown that bacteria can be targeted using aggregation-induced emission fluorogens (AIEgens) Mito-triphenylphosphonium (TPP) a lipophilic cation (155) that disrupt mitochondrial functions of bacteria as well cancer cells (156). TPPCN, a multifunctional luminogen, with the ability to generate reactive oxygen species (ROS) can cause damage to both cancer cells and bacteria (156). Thus, agents like TPPCN and other novel agents may be utilized to develop robust pre-clinical models of targeting microbiota-induced stemness, and whether such agents would help us to aid in targeted as well as immunotherapy needs to be explored.

## Conclusion

OSCC are highly heterogeneous, immunosuppressive and highly aggressive tumor. The interaction of oral microbiota with the precancerous lesions of the oral cavity may contribute to OSCC initiation and progression. The inflammatory microenvironment of OSCC may be enhanced by specific oral microbiota such as HPV, PG and FN. These and yet unidentified oral microbiota species may induce stemness in oral cancer cells. Importantly, oral microbiota may modulate CSC niche defense by activating tumor stemness pathways, as well as modulating epigenetic mechanism. Therefore it is important to develop novel *in vitro* and *in vivo* models to study interaction between oral microbiota and oral cancer cells/CSCs that leads to the enhancement of CSC stemness including the induction of TSD phenotype. Overall, novel approaches are needed to study the complex interaction between the oral microbiota and cancer stemness to identify new targets against OSCC progression and relapse.

## Author contributions

BD has conceptualized and edited the article. PS has written, edited, proofread the article and also created the figures and table. LP has written and edited the article. SM has written and proofread the article. All authors contributed to the article and approved the submitted version.
